# TETRAS Spirals and Handwriting Samples: Determination of Optimal Scoring Examples

**DOI:** 10.5334/tohm.665

**Published:** 2021-11-16

**Authors:** William G. Ondo, Aparna Wagle Shukla, Carson Ondo, On behalf of the Tremor Research Group

**Affiliations:** 1 Methodist Neurological Institute, Houston, TX, US; 2 Weill Cornell Medical School, US; 3 University of Florida, US; 4 St Thomas H.S., US

**Keywords:** tremor, spiral, writing, TETRAS

## Abstract

**Background:**

Spiral drawings and handwriting tasks have long been used to assess the severity of essential tremor, but these motor tasks are somewhat less objective as the rules for scoring are not based on firm objective amplitude-based criteria. Publishing the best examples of each of the possible 0–4 ratings for these items could reduce scoring variance.

**Methods:**

21 members of the Tremor Research Group each rated 94 spirals and 64 handwriting samples using TETRAS scoring criteria. For each sample, the most frequently reported score (mode; maximum of 21) was determined. Ratings not adjacent to the mode were subtracted from the number of mode scores, to calculate a total value. For each of the ratings (0, 1, 1.5, 2, 2.5, 3, 3.5, 4), the samples with the highest total value were selected as best examples.

**Results:**

In general, rater agreement was good for spirals but poor for handwriting samples. Nevertheless, examples with excellent agreement were identified for all spiral and handwriting ratings, and are presented.

**Conclusion:**

Best examples for scoring spirals and handwritings are needed to reduce the variance of TETRAS scores in clinical trials and clinical practice.

The Tremor Research Group Essential Tremor Assessment Scale (TETRAS) is a well-validated performance (examination) and activity of daily living scale designed specifically for essential tremor [1]. Many of the performance tasks in TETRAS are similar to preceding scales but the instructions are more explicit, objective and codified. The anchors for 0–4 ratings (0, 1.0, 1.5, 2.0, 2.5, 3.0, 3.5, 4.0) are designed to minimize the floor and ceiling effects, yet remain sensitive to treatment effect.

During the original validation studies of the TETRAS, we found greater scoring variance for writing and spirals compared to arm posture, wing-beating, and kinetic assessments, which all have objective amplitude ranges, not possible for writing and spirals. [1, personal communication] The scoring instructions for the spiral drawing task include: none (0), slight: barely visible (1) mild: obvious (2), moderate: portions not recognizable (3), severe: figure not recognizable (4), which is highly subjective. Similarly, the instructions for the handwriting task include: none (0), slight: untidy (1), mild: legible but considerable tremor (2), moderate: parts illegible (3), severe: completely illegible (4). Furthermore, the use of 0.5 point increments in ratings is encouraged if there is uncertainty between two “defined” integers, e.g. 1.5 if between mild (1) and moderate (2). In the original validation studies however non-integer values were not utilized by more than 50% of raters. Besides the validation studies, data from recent clinical trials revealed a similar variance in the scores (personal communication) prompting the study sponsors to request examples of the “correct” scoring for these items. Therefore, we sought to examine the score distribution for spirals and handwritings among a group of trained tremor specialists. The goal was to identify the best examples for each of the 0–4 ratings for these items based on a high level of agreement among the raters.

## Methods

Spirals and writing samples were obtained from the clinic of one of the authors (W.O.) as part of the TETRAS, which is done for all ET patients, and as standard of care was IRB exempt. The samples were originally written with a ball point pen on paper, following TETRAS instructions. Spiral drawings were unsupported (not touching surface), the pen could be gripped anywhere, spirals were drawn with 4-5 revolutions about 1 cm apart, which should cover ¼ of a standard piece of paper. Direction of the spiral is not specified. Instructions for writing were simply to write “This is a sample of my best handwriting” normally, only with the dominant hand, in cursive unless unable to write cursive, in which case print writing is allowed. Samples at least partially written in cursive were used. Many samples contained a mix of cursive and print. Ninety-four spiral drawings and 64 writing samples reflecting the entire range of possible ratings on TETRAS were collected. Spiral and writing samples were scanned into PDF files. These were then isolated in photoshop and transferred to Google forms, which were subsequently distributed to Tremor Research Group (TRG) members for rating.

Participating raters were provided scoring instructions and a pictorial guide of examples for each score (as determined by W.O.) to use “as they see fit”. Participating raters were encouraged to use 0.5 point increments in scoring in a continuum with integers in order to create an interval, rather than just ordinal scale. Since the goal of this study is to present the most agreed upon examples of each rating for publication, rather than other metric evaluations, we determined the mode (maximum 21) for each sample. We then subtracted any responses rated for scores that were not immediately adjacent to the mode score, in order to calculate the total score. We considered the sample with the highest total value as the best example. For example, if in a sample of 12 responses (1,1.5, 2, 2, 2, 2, 2, 2, 2, 2.5, 2.5, 3, 3); the score of 2 is the mode with 7 responses minus the 3 response for scores not adjacent to the mode (1, 3, 3) will lead to a total score of 4. In case of a tie, we used the example score closest to the mean score. Two spiral and one writing sample are presented for each score.

## Results

**Figure 1:**
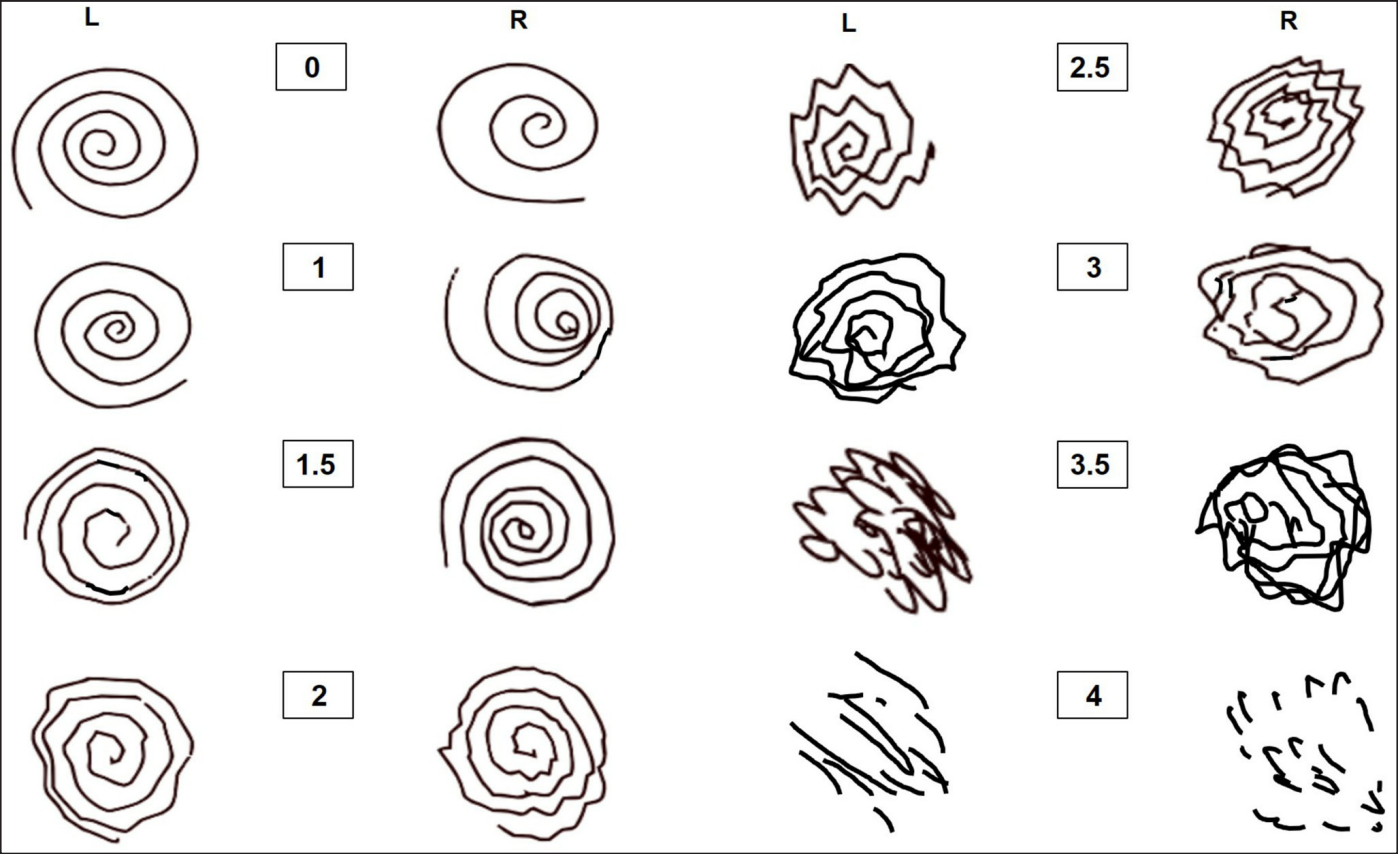
Two spiral samples for each score with the highest scoring agreement.

**Figure 2:**
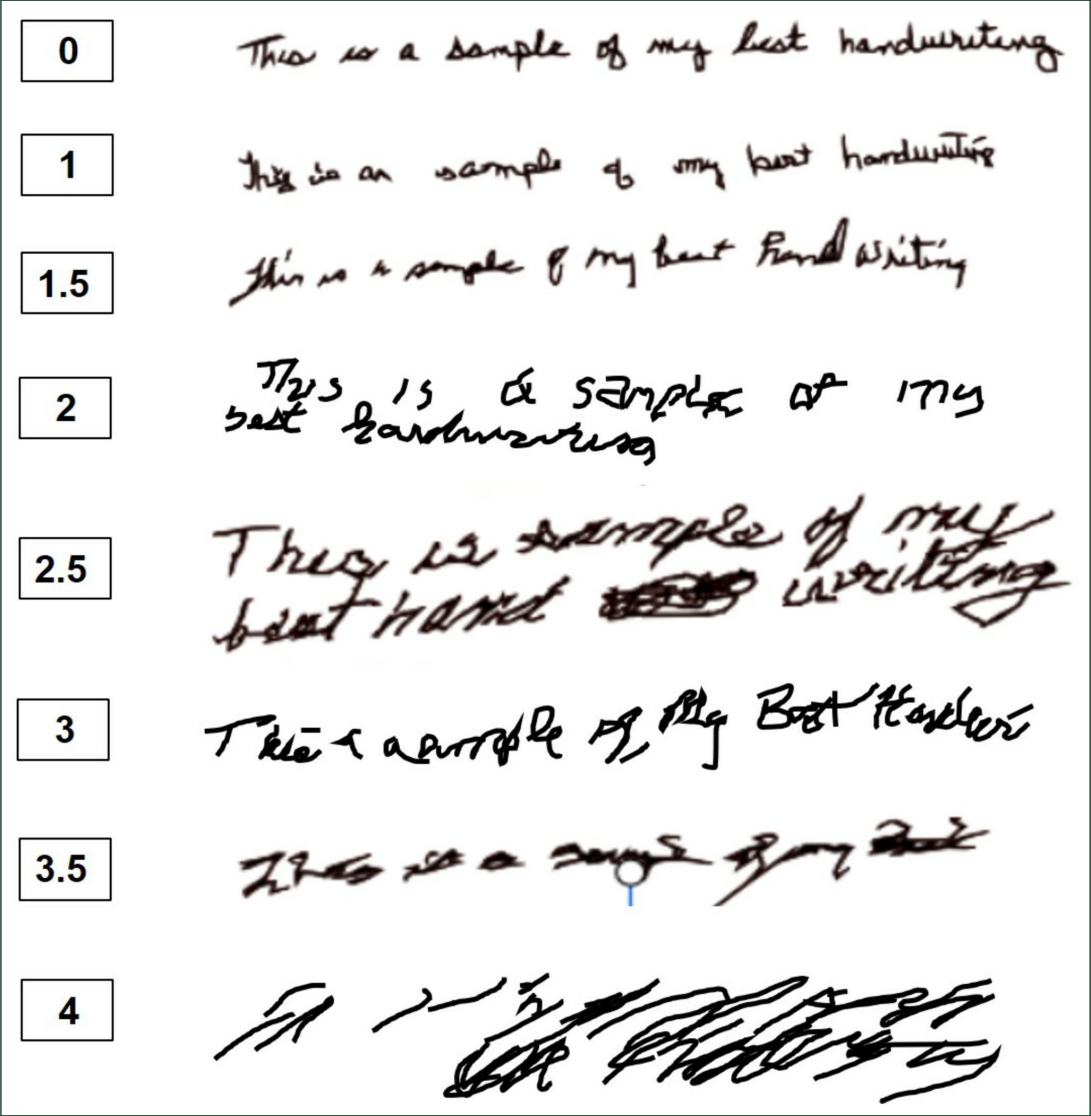
One writing example for each score with the highest scoring agreement.

Twenty-one TRG members each rated 94 spirals and 64 handwriting samples. Based on total value calculation, the best examples identified for each rating are presented in ***Figure 1*** (spirals) and ***Figure 2*** (handwriting).

For the spirals, the total number of different responses was 1 (perfect agreement) in 4 samples, 2 different responses in 18 samples, 3 different responses in 41 samples, 4 different responses in 23 samples, 5 different responses in 7 samples, and 6 different responses in one sample (Supplementary Table 1). Therefore 68% of the 95 spiral samples had 3 or fewer responses. The number of modes for each rating was: 0 (6), 1 (19), 1.5 (17), 2 (16), 2.5 (14), 3 (8), 3.5 (10), 4 (4).

For the handwriting, the total number of different responses was 1 (perfect agreement) in 4 samples, 2 different responses in 7 samples, 3 different responses in 9 samples, 4 different responses in 15 samples, 5 different responses in 13 samples, 6 different responses in 14 samples, and 7 different responses (out of a possible 8) in 2 samples (Supplementary Table 2). Therefore only 31% had 3 or less responses. Despite this, we did identify at least 1 example of each score with good agreement, as defined by a score >50% of the maximum. All cases with perfect agreement from all 21 raters were either a 0 or 4 rating. The number of modes for each rating was: 0 (9), 1 (15), 1.5 (8), 2 (9), 2.5 (4), 3 (9), 3.5 (3), 4 (6).

In all examples that were finally selected, the score based on the mode value equaled the median score and was quite close to the mean score (<0.14 points from mean).

## Discussion

We present spiral drawing and handwriting examples which demonstrate appropriate TETRAS scoring based on identifying examples with the greatest rater agreement among tremor experts, picked from a large group of possible examples. The relatively poor agreement in scoring, even among tremor experts, highlights the need for published examples to help guide ratings, especially for writing samples.

A few points warrant further consideration. Poorer agreement on writing samples was expected given the complexity and greater natural non-tremor related variability of writing, compared to spirals. Scores for writing examples with particularly poor agreement (N = 8) usually ranged from 0 to 3 (Supplemental Figure 1). Post-rating comments from raters suggested that some looked more for overt line oscillations whereas others were influenced by general sloppiness.

The TETRAS writing samples and spirals both have 8 possible scoring options. The best number of options for a scale is debatable. General psychometrics often advocate 5 or 7 options, but this clearly depends on the perceptible scope of the task, and we feel 8 options are clearly discernible with spirals and probably discernable with writing samples. The Bain and Findley spiral assessments, for example, created and sampled 10 spiral scoring options based on the median scores of 4 raters [2]. We suspect that including 0.5 increments in our scale for a total of 8 options, compared to 5 options, increases sensitivity to treatment effect. However, proving this for this specific scenario would require a prospective direct comparison study, which is not planned.

TETRAS spirals are drawn freehand, rather than on a template, (in between lines or traced on top of a spiral). This was decided based on a study that found better inter-rater concordance with freehand writing [3]. Also freehand has the advantage of not requiring any template. Overall, the superior rater correlations of spirals vs. writing, argues that spirals are the better single rating measure. Spirals also evaluate both hands instead of one.

The current writing scoring is done only in English. It is not known whether this could accurately guide writing in other languages. We only included mostly cursive samples, as per TETRAS instructions. Previously, the TRG reported that cursive writing is rated slightly worse than print, but there is a very strong correlation [4]. As cursive is no longer taught in many places, eventually similar rating examples for print will be required.

Samples drawings scored by W.O. were provided to the group as a guide “to use as they see fit”. This was done to emphasize the non-integer scores and because the original validation studies only provided written instructions (slight, mild, moderate, severe), and it was felt that an intermediate step was needed to narrow the potential scope for raters prior to creating a definitive selection of examples. This could have introduced bias to the group to pick those examples, but only 2/8 writing samples and 5/16 spiral examples were chosen that were identical to the provided samples.

We feel that these examples should improve scoring consistency for these portions of the TETRAS performance scale when used clinically, and especially when used in clinical trials. Furthermore, regulatory agencies have emphasized the writing portion of the TETRAS over the postural-wing-beating-kinetic arm portion because writing is an activity of daily living, whereas those “artificial” positions are not (personal communication). Likewise, accelerometry based assessments, which do correlate well with the TETRAS postural-wing-beating-kinetic arm assessments [5], have been even further de-emphasized and are unlikely to be approved as a primary efficacy point in the foreseeable future (personal communication).

## Summary

We present best TETRAS scoring examples of spiral and handwriting using an objective formula based on ratings of tremor experts. This should improve reliability of TETRAS scoring.

## Additional Files

The additional files for this article can be found as follows:

10.5334/tohm.665.s1 Supplemental Table 1. All spiral data.

10.5334/tohm.665.s2 Supplemental Table 2. All writing data.

10.5334/tohm.665.s3 Supplemental Figure 1. Writing sample with poor agreement.

## References

[1] Elble R, Comella C, Fahn S, et al. Reliability of a new scale for essential tremor. Mov Disord. 2012; 27: 1567–1569. DOI: 10.1002/mds.25162 23032792 PMC4157921

[2] Bain P, Findley L, Atchison P, et al. Assessing Tremor Severity. J Neuro Neurosurg Psych. 1993; 56: 868–73. DOI: 10.1136/jnnp.56.8.868 PMC1015140 8350102

[3] Ondo WG, Wang A, Thomas M, Vuong K. Assessing Factors That Can Influence Spirograph Performance in Patients with Essential Tremor. Park Dis Rel Disord. 2005; 11: 45–48. DOI: 10.1016/j.parkreldis.2004.07.005 15619462

[4] Ondo WG, Pascual B, On behalf of the Tremor Research Group. Tremor Research Group Essential Tremor Rating Scale (TETRAS): Assessing Impact of Different Item Instructions and Procedures. Tremor and Other Hyperkinetic Movements. 2020; 10: 36. DOI: 10.5334/tohm.64 33101762 PMC7546108

[5] Mostile G, Giuffrida J, Adam A, et al. Correlation between Kinesia system assessments and clinical tremor scores in patients with essential tremor. Mov Disord. 2010; 25: 1938–43. DOI: 10.1002/mds.23201 20623687

