# Supplementary material for: TETRAS Spirals and Handwriting Samples: Determination of Optimal Scoring Examples
Source: Tremor Other Hyperkinet Mov (N Y). 2021 Nov 16;11:50. doi: 10.5334/tohm.665 (PMC8603854; doi:10.5334/tohm.665)
Supplement: Supplemental Figure 1. — Writing sample with poor agreement. [file tohm-11-1-665-s3.pdf]

This is a sample of my Best Hand writing	0-3
This is a sample of my best handwriting	0-3
This is a sample of my best handwriting	0-3
This is a sample of my best handwriting	0-3
This is a sample of my best handwriting	0-3.5
This a sample of my beat handwriting	0-3
This is a sample of my best handwriting.	0-3.5
This is a sample of my best handwriting	0-3
